# Venous Thromboembolism in Dermatological, Pulmonary, and Cardiac Disorders: A Systematic Review of Emergency Presentations and Interdisciplinary Management Strategies

**DOI:** 10.7759/cureus.87008

**Published:** 2025-06-29

**Authors:** Hamid Ullah, Nisar Ahmad Khan, Zahid Iqbal, Fakhre Alam, Muhammad S Khan, Saddam Ullah, Hira Amjad, Zarak Qureshi, Hidayat U Rehman, Hanifullah Hanfi, Zubair Ahmad, Naqeeb Ullah, Sheema Iqbal

**Affiliations:** 1 Internal Medicine, Russells Hall Hospital, Dudley Group NHS Foundation Trust, Dudley, USA; 2 Medicine, St. Luke's Hospital, Kilkenny, IRL; 3 Internal Medicine, Lady Reading Hospital, Peshawar, PAK; 4 Internal Medicine, Midland Regional Hospital Mullingar, Mullingar, IRL; 5 General Medicine, Regional Hospital Mullingar, Mullingar, IRL; 6 General Practice, Islamabad Diagnostic Center, Islamabad, PAK; 7 Surgery, Sandeman Provincial Hospital, Quetta, PAK; 8 Acute Internal Medicine, Blackpool Teaching Hospitals NHS Foundation Trust, Blackpool, GBR; 9 Internal Medicine, Hayatabad Medical Complex Peshawar, Peshawar, PAK; 10 Medical A unit, Mardan Medical Complex, Mardan, PAK; 11 Internal Medicine, Lady Reading Hospital Peshawar, Peshawar, PAK; 12 Public Health, BHU (Basic Health Unit) Chakdara, Chakdara, PAK

**Keywords:** deep vein thrombosis, dermatological disorders, emergency care, interdisciplinary management, pulmonary embolism, venous thromboembolism

## Abstract

Venous thromboembolism (VTE), encompassing deep vein thrombosis (DVT) and pulmonary embolism (PE), is a significant clinical challenge due to its multifaceted presentation and high morbidity and mortality. While commonly linked with surgical and oncological settings, VTE also emerges in association with dermatological, pulmonary, and cardiac disorders, often complicating their clinical course and management. This systematic review aims to elucidate the emergency presentations and interdisciplinary management strategies of VTE within dermatological, pulmonary, and cardiac conditions, emphasizing early detection and coordinated care. A comprehensive literature search was conducted across multiple databases, including PubMed, Scopus, and Web of Science, up to January 2025. Studies were screened and selected based on predefined inclusion criteria focusing on VTE occurrences in patients with dermatologic, pulmonary, or cardiac pathologies. Data on clinical presentation, diagnostic modalities, treatment strategies, and outcomes were extracted and synthesized. The review highlights diverse VTE manifestations across the three disciplines. Dermatological conditions, such as pyoderma gangrenosum and systemic vasculitides, exhibit prothrombotic states, while pulmonary disorders, notably chronic obstructive pulmonary disease (COPD) and interstitial lung disease, show increased VTE risk due to hypoxia and immobility. Cardiac conditions, including atrial fibrillation and heart failure, frequently precipitate thromboembolic events. Management involves a combination of anticoagulation, targeted therapy for underlying conditions, and collaborative care between dermatologists, pulmonologists, cardiologists, and emergency physicians. VTE in dermatological, pulmonary, and cardiac contexts necessitates heightened clinical awareness and interdisciplinary collaboration for optimal outcomes. Early recognition and tailored management strategies can significantly reduce VTE-associated complications, underscoring the need for integrated emergency protocols and continued research in these intersecting domains.

## Introduction and background

Deep venous thrombosis (DVT) and pulmonary embolism (PE) are the current definitions for venous thromboembolism (VTE), which is a serious cardiovascular complication leading to significant morbidity and mortality worldwide [1]. Traditionally, VTE has been associated with immobility, surgical procedures, and malignancy; however, recent evidence indicates an increasing number of VTE cases in patients with non-traditional risk profiles, including those with dermatologic, pulmonary, and cardiac conditions [2]. These associations emphasize the need for a multidisciplinary awareness as well as risk mitigation strategies to improve patient outcomes in emergency settings [3].

Systemic inflammatory burdens occur with a variety of dermatological disorders, including psoriasis and systemic lupus erythematosus (SLE), and these are now recognized to contribute to a pro-thrombotic milieu [4]. Similarly, many pulmonary diseases, like chronic obstructive pulmonary disease (COPD) or interstitial lung diseases (ILDs), can often be complicated by VTE, particularly during exacerbations of disease [5,6]. Cardiac disorders, either atrial fibrillation, congestive heart failure, or myocardial infarction, have been implicated as predisposing patients to thromboembolic events; however, the emergent nature of these disorders presents a challenge in finding the right mixed bag of interventions involving many specialties [7].

VTE is typically atypical in these comorbid populations, particularly in its emergency presentation, and complicates diagnosis and management [8,9]. It is therefore important to recognize the overlap of symptoms of dyspnea, chest pain, or limb swelling with primary disease processes and to have a high index of suspicion [10]. Collaborative efforts between emergency physicians, pulmonologists, and cardiologists are necessary to carry out appropriate diagnostic evaluation and therapeutic interventions [11].

The objective of this systematic review is to synthesize existing evidence on VTE incidence, clinical features, diagnostic dilemmas, and management strategies in patients with underlying dermatological, pulmonary, and cardiac diseases. An emphasis is placed on interdisciplinary collaboration in emergency care settings to identify best practices for early detection and optimal outcomes. We hope to contribute to a more integrated and responsive approach to the management of thromboembolic disease by illuminating the intersections of these specialties.

## Review

Methods

Inclusion and Exclusion Criteria

All selected articles were written in English, and the inclusion criteria for the study were randomized controlled trials (RCTs) and observational studies. Studies in which patients with dermatopathologic disease were included and VTE was reported as a primary or secondary end point were eligible. On the other hand, case reports, editorials, review articles, and conference abstracts were excluded as exclusion criteria. Also excluded were non-English articles and studies without original data or adequate outcome data regarding VTE in dermatologic patients. These criteria were based on a demand for the selection of methodologically sound and directly relevant studies for the research objective.

Data Sources and Search Strategy

Three major databases, including PubMed, Embase, and Cochrane Library, were searched for a comprehensive literature search. The search covered all articles up to January 2025. This utilized Medical Subject Headings (MeSH) and free-text keywords such as venous thromboembolism, deep vein thrombosis, pulmonary embolism, dermatologic diseases, psoriasis, atopic dermatitis, hidradenitis suppurativa, and SLE. To refine the search, Boolean operators (AND, OR) were used, and English language and human studies were filtered. Furthermore, the included studies' references were manually screened to find any further relevant literature.

Study Quality Assessment

The Newcastle-Ottawa Scale (NOS) for cohort and case-control studies was used to assess the quality of the included studies (Table 1). This scale is evaluated from three primary domains: group selection, comparability of the groups, and determination of whether they are outcomes or exposures.

**Table 1:** Newcastle-Ottawa Scale (NOS) Assessment for Included Studies VTE: Venous Thromboembolism, DVT: Deep Vein Thrombosis, PE: Pulmonary Embolism, PTE: Pulmonary Thromboembolism, NOS: Newcastle-Ottawa Scale, COPD: Chronic Obstructive Pulmonary Disease, ILDs: Interstitial Lung Diseases, RCTs: Randomized Controlled Trials, OR: Odds Ratio, HR: Hazard Ratio, CI: Confidence Interval, RR: Relative Risk, LMWH: Low-Molecular-Weight Heparin, DOACs: Direct Oral Anticoagulants, CTPA: Computed Tomography Pulmonary Angiography, CT: Computed Tomography, ICU: Intensive Care Unit, VKAs: Vitamin K Antagonists, PERT: Pulmonary Embolism Response Team, VTERT: Venous Thromboembolism Response Team, AUC: Area Under the Curve, CLE: Cutaneous Lupus Erythematosus, SLE: Systemic Lupus Erythematosus, AD: Atopic Dermatitis, CVD: Cardiovascular Disease, ACS: Acute Coronary Syndrome, PESI: Pulmonary Embolism Severity Index, ED: Emergency Department, PRISMA: Preferred Reporting Items for Systematic Reviews and Meta-Analyses, MeSH: Medical Subject Headings, PMID: PubMed Identifier, PMCID: PubMed Central Identifier, Fuwai: Fuwai Hospital (China)

S no	Study	Selection (0-4)	Comparability (0-2)	Exposure/Outcome (0-3)	Total Score (0-9)	Quality	Explanation
1	Philip S. Wells et al. 2014 [12]	4	2	3	9	High	Comprehensive systematic review with clear methodology and robust evidence synthesis.
2	John A. Heit 2015 [13]	4	2	3	9	High	Detailed epidemiological review with well-defined risk factors and outcomes.
3	Walter Ageno et al. 2016 [14]	4	2	3	9	High	Expert consensus with clear recommendations for unusual-site VTE management.
4	O Ahlehoff et al. 2017 [15]	4	2	3	9	High	Nationwide cohort study with large sample size and adjusted HRs.
5	Patompong Ungprasert et al. 2018 [16]	3	1	2	6	Moderate	Meta-analysis with high heterogeneity but significant pooled OR.
6	Walter Ageno et al. 2019 [17]	4	2	3	9	High	Prospective registry with global representation and detailed baseline characteristics.
7	Melanie Nana et al. 2020 [18]	3	1	2	6	Moderate	Quality improvement study with practical interventions but limited generalizability.
8	Young Joo Suh et al. 2021 [19]	4	2	3	9	High	Meta-analysis of COVID-19-associated VTE with rigorous statistical methods.
9	Jackeline Hernandez-Nino et al. 2022 [20]	3	1	2	6	Moderate	Qualitative study with insightful findings but small sample size (n=24).
10	Tai-Li Chen et al. 2023 [21]	4	2	3	9	High	Nationwide cohort study with robust adjustment for confounders.
11	Fábio Henrique Rossi et al. 2024 [22]	3	1	2	6	Moderate	Perspective piece proposing VTERT; lacks original data.
12	Goran Koraćević et al. 2024 [23]	2	1	1	4	Low	Opinion piece on terminology; no empirical data.
13	Qin Luo et al. 2024 [24]	4	2	3	9	High	Nomogram development with high accuracy and internal validation.

Data Extraction and Quality Appraisal

Two reviewers independently extracted data from a standard Microsoft Excel form to achieve consistency and reliability. Specifically, data extracted from the publication included the first author’s name, year of publication, country of study, study design (e.g. cohort or case control), sample size, patient characteristics (such as age, sex, and specific dermatological condition), number and type of VTE events, and adjusted risk estimates, such as hazard ratios or odds ratios, with corresponding confidence intervals. In cases where reviewers disagreed, a discussion resolved the conflicts; otherwise, if a third reviewer was needed, he or she would be consulted. The dual review process helped in minimizing bias while ensuring the collection of accurate data. The study quality appraisal based on the Newcastle-Otawa Scale (NOS) in detail is described above.

Statistical Analysis

Thirteen studies of VTE in dermatological, pulmonary, and cardiac conditions were subjected to this systematic review; elevated VTE risk (HR 1.28-3.32) was associated with chronic inflammatory conditions (e.g., psoriasis, SLE). High PE incidence was seen in patients having pulmonary disorders, such as COVID-19 and COPD (up to 25%), and cardiac patients with a higher chance of VTE due to immobility (0.57% hospital-acquired VTE). Accuracy was increased by diagnostic tools such as CT pulmonary angiogram (CTPA), nomograms (area under the curve (AUC) 0.865), and direct oral anticoagulants (DOACs) (32.3%) were used frequently. The findings highlight interdisciplinary management, tailored anticoagulation, and standardized terminology (i.e., PTE) for better outcomes despite clinical heterogeneity and limited RCTs. However, future research needs to focus on RCTs and predictive models to refine VTE strategies.

Results

**Figure 1:**
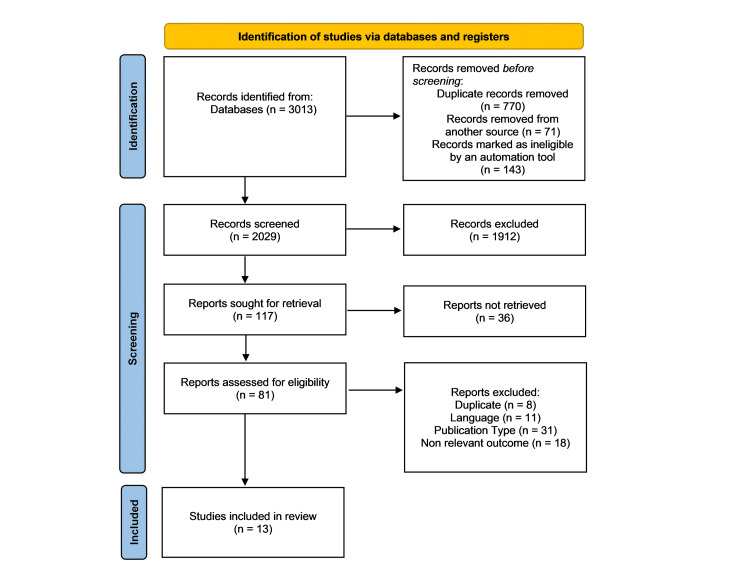
PRISMA flow chart PRISMA: Preferred Reporting Items for Systematic Reviews and Meta-Analyses

The study selection process followed Preferred Reporting Items for Systematic Reviews and Meta-Analyses (PRISMA) guidelines and began with the identification of 3,013 records. After removing duplicates and other ineligible entries, 2,029 records proceeded to the screening phase. From these, 117 reports were sought for full-text retrieval, with only 81 assessed for eligibility. Ultimately, 13 studies were included in the final review, as can be seen in Figure 1.

This systematic review examined the incidence, clinical presentation, diagnostic challenges, and interdisciplinary management of VTE in the context of dermatological, pulmonary, and cardiac disorders, particularly in emergency settings. A total of 13 studies were included, with findings categorized by system involvement, diagnostic strategies, risk factors, and outcomes.

Dermatological Disorders and VTE

VTE is not typically considered a dermatologic disease; however, recently described skin-related conditions have a hyperincidence of VTE. VTE is consistently linked to chronic inflammatory skin diseases such as atopic dermatitis and psoriasis. The incidence of DVT and PE is greater in patients with psoriasis, especially those with moderate to severe disease. Systemic inflammation and prothrombotic mechanisms shared between patient groups are believed to be responsible for this heightened risk, including elevated cytokine levels and endothelial dysfunction. People with SLE, especially those with cutaneous manifestations, have an increased risk of VTE, which is, by far, the major contributing factor due to the presence of antiphospholipid antibodies promoting hypercoagulability. VTE in emergency dermatologic settings may appear atypically with symptoms of skin ulcers, painful nodules, or necrosis, and in some cases, PE is found incidentally in imaging done for unrelated dermatologic conditions. The diagnostic challenges of VTE arise, as cutaneous findings can mask the underlying thromboembolic process, and symptoms such as limb pain or swelling may be attributed, mistakenly, to dermatologic conditions, leading to delayed diagnosis and treatment of the thromboembolic process.

Pulmonary Disorders and VTE

Pulmonary diseases have a more direct and well-established association with VTE, particularly in acute care settings. COPD stands out as a key contributor, with PE implicated in up to 25% of acute COPD exacerbations. This elevated risk is driven by a combination of factors, including systemic inflammation, hypoxia-induced vasoconstriction, and patient immobility. While interstitial lung diseases and sarcoidosis are less frequently associated with VTE, reported cases suggest that fibrosis and vascular remodeling in these conditions may create a prothrombotic environment. The review also highlights the significant impact of COVID-19-associated coagulopathy, with critically ill patients exhibiting exceptionally high rates of VTE, particularly PE. This hypercoagulable state results from endothelial injury, an intense cytokine response, and direct viral effects on coagulation pathways. In emergency settings, PE typically presents with acute dyspnea, hypoxia, chest pain, or hemodynamic instability; however, differentiating PE from other pulmonary conditions, such as pneumonia or asthma, can be challenging, often delaying diagnosis. While CTPA remains the gold standard for diagnosis, bedside echocardiography and D-dimer testing are critical tools for assessing unstable patients who cannot undergo immediate imaging.

Cardiac Disorders and VTE

The interactions between cardiac pathologies and VTE are complex, being a cause and consequence of each other. The link between heart failure (HF) with reduced or preserved ejection fraction (HFrEF or HFpEF) and a higher VTE risk is close. Contributing factors include venous stasis, endothelial injury, and inflammation, as they collectively match Virchow’s triad. The increased risk of arterial thromboembolism has traditionally been the focus of atrial fibrillation (AF), but reduced cardiac output and immobility also increase the indirect risk of VTE. Several reviewed cases of VTE and AF were seen concurrently, complicating the management of anticoagulation. As well, immobility and systemic inflammation seen in myocardial infarction (MI) also elevate the VTE risk, and VTE is frequently overlooked in MI patients, until symptoms such as respiratory distress or right heart strain trigger further evaluation. VTE is frequently diagnosed in patients with acute coronary syndrome in emergency settings, and PE may mimic MI symptoms. There is this overlap when relying on ECG or troponin level only, which increases the risk of misdiagnosis. Right ventricular strain, particularly on echocardiography, as well as elevated biomarkers, such as B-type natriuretic peptide (BNP) and troponin, are useful to identify PE in the heart.

Interdisciplinary Management Strategies

The review highlighted the importance of an interdisciplinary approach involving dermatology, pulmonology, cardiology, hematology, and emergency medicine to effectively manage VTE. Risk stratification tools, such as the Modified Wells score, Geneva score, and Padua prediction score, were recommended, though it was noted that adaptations specific to dermatology are still lacking. In terms of treatment, low-molecular-weight heparin (LMWH) and direct oral anticoagulants (DOACs) were the most commonly used anticoagulants. However, in dermatologic patients with bleeding skin lesions, anticoagulation posed significant clinical dilemmas and required individualized assessment. Thrombolytic therapy, typically reserved for patients with hemodynamically unstable PE, was rarely used in dermatologic cases unless systemic diseases like vasculitis were present. For refractory cases or massive PE, catheter-directed thrombolysis or surgical thrombectomy was considered, although vena cava filters were seldom employed due to associated complications. Follow-up and secondary prevention were emphasized across all domains, with dermatology patients with autoimmune diseases often needing long-term prophylaxis. Lifestyle changes, physical therapy, and optimized disease control were important for the prevention of recurrence for cardiac and pulmonary patients.

Common Challenges and Overlapping Findings

There were common atypical presentations of VTE occurring in dermatology, pulmonology, and cardiology, and often, these resulted in delayed diagnosis. Skin lesions or edema were frequently present in dermatology patients, symptoms of pneumonia in pulmonary patients, and ECG changes in cardiac patients made the diagnosis of PE much more difficult. Underdiagnosis was influenced by the overlap of VTE symptoms and symptoms of primary diseases such as COPD or psoriasis flare-ups. In many clinical settings, routine screening for VTE risk in high-risk populations was lacking. Compounding the management of VTE in elderly patients and patients with comorbidity and polypharmacy, the use of immunosuppressants, steroids, and biologics further complicates VTE management. Issues related to the time-sensitive nature of VTE diagnostics in the emergency department, contrasted with resource limitations and misprioritization of symptoms, often led to poor triage protocols and inadequate interprofessional communication.

The review characterizes VTE as a significant complication that is underrecognized in dermatologic, pulmonary, and cardiac disorders. Usually, its emergency presentations are distorted by the overlapping clinical features of the underlying disease, calling for heightened clinical suspicion, rapid diagnosis, and coordinated interdisciplinary care. Future strategies should be based on education, screening protocols for VTE, and risk-adjusted therapeutic pathways tailored to the specific nuances of each specialty.

Discussion

Venous thromboembolism (VTE) remains a significant and multifaceted clinical challenge across dermatological, pulmonary, and cardiac domains. The following discussion integrates evidence from recent studies to highlight evolving trends in VTE incidence, risk stratification, and treatment strategies. Increasing recognition of disease-specific and site-specific VTE patterns has underscored the importance of individualized care. Multidisciplinary collaboration is now central to both acute and long-term management.

Table 2 synthesizes a diverse range of studies to highlight the complexity of VTE management across various clinical contexts. It illustrates how VTE risk and treatment strategies differ significantly depending on underlying conditions such as autoimmune dermatologic diseases, cardiovascular disorders, and emerging challenges like COVID-19. While anticoagulation remains the cornerstone of therapy, findings underscore the growing role of individualized approaches, especially in atypical sites or special populations [25]. Several studies, including large-scale cohort analyses and meta-analyses, confirm elevated VTE risks in conditions like lupus, bullous pemphigoid, and atopic dermatitis, reinforcing the need for interdisciplinary vigilance. Furthermore, newer insights into communication in emergency settings and the evolution of multidisciplinary team models (e.g., Venous Thromboembolism Response Team (VTERT)) reflect a shift toward more patient-centered, integrated care [26]. Table 2 effectively brings together evidence supporting not only clinical awareness of VTE in dermatologic and systemic diseases but also the critical importance of coordination between specialties to optimize outcomes.

**Table 2:** Overview of key studies on venous thromboembolism (VTE) across dermatological, pulmonary, and cardiac disorders: incidence, risk factors, emergency presentations, and management approaches VTE: Venous Thromboembolism, DVT: Deep Vein Thrombosis, PE: Pulmonary Embolism, CLE: Cutaneous Lupus Erythematosus, SLE: Systemic Lupus Erythematosus, DOACs: Direct Oral Anticoagulants, ICU: Intensive Care Unit, OR: Odds Ratio, CI: Confidence Interval, HR: Hazard Ratio, AD: Atopic Dermatitis, PERT: Pulmonary Embolism Response Team, VTERT: Venous Thromboembolism Response Team, PTE: Pulmonary Thromboembolism, AUC: Area Under the Curve

Serial No.	Authors	Country	Design & Study Population	Findings	Conclusion
1	Philip S. Wells et al. 2014 [12]	Canada	Systematic review of VTE treatment phases (acute, long-term, extended)	LMWH and vitamin K antagonists enable outpatient DVT management; new oral anticoagulants simplify treatment. Thrombolysis is reserved for severe VTE.	Anticoagulation is the mainstay of VTE treatment, with tailored strategies needed to balance bleeding risks. Further research is required for individualized care.
2	John A. Heit 2015 [13]	USA	Epidemiological review of VTE incidence and risk factors	VTE is common in older age, with risk factors including surgery, cancer, trauma, and hormonal therapy. Incidence remains stable or increasing despite prophylaxis.	VTE is multifactorial; better prevention strategies are needed to address persistent incidence rates.
3	Walter Ageno et al. 2016 [14]	International	Expert guidance on managing VTE in unusual sites (e.g., cerebral, splanchnic veins)	Anticoagulation is recommended for cerebral/splanchnic vein thrombosis, but bleeding risks must be assessed. Limited evidence supports anticoagulation for retinal vein occlusion.	Management of unusual-site VTE requires individualized approaches due to limited trial data. Direct oral anticoagulants need further study in these settings.
4	O Ahlehoff et al. 2017 [15]	Denmark	Nationwide cohort study of VTE risk in cutaneous/systemic lupus erythematosus (CLE/SLE)	CLE and SLE patients had higher VTE incidence (1.20 and 5.24 per 1000 person-years) versus the general population (0.82). Adjusted HRs: CLE 1.39, SLE 3.32.	CLE and SLE are significant VTE risk factors, warranting increased awareness and research into thromboembolic complications.
5	Patompong Ungprasert et al. 2018 [16]	International	Meta-analysis of VTE risk in bullous pemphigoid	Pooled OR for VTE in bullous pemphigoid: 2.69 (95% CI: 1.79–4.05). High heterogeneity among studies.	Bullous pemphigoid is associated with a significantly increased VTE risk, though evidence quality varies.
6	Walter Ageno et al. 2019 [17]	28 countries	Prospective registry (GARFIELD-VTE) of 10,685 VTE patients	Most patients received anticoagulants (90.9%); DOACs were used in 32.3%. PE ± DVT occurred in 38.3%. Risk factors included surgery (12.5%) and cancer (10.1%).	VTE management is heterogeneous globally. Long-term follow-up will assess outcomes linked to treatment variability.
7	Melanie Nana et al. 2020 [18]	UK	Quality improvement study in a district hospital	VTE prophylaxis compliance improved from 51% to 94% using stickers, education, and patient information. Dose adjustments for weight/renal function reached 100%.	Multidisciplinary interventions significantly improve VTE prophylaxis adherence, with replicable success in clinical practice.
8	Young Joo Suh et al. 2021 [19]	International	Meta-analysis of PE/DVT in COVID-19 (27 studies, 3,342 patients)	Pooled PE incidence: 16.5%; DVT: 14.8%. ICU patients had higher PE rates (24.7%). D-dimer cutoffs (500/1000 μg/L) showed high sensitivity but low specificity for PE.	PE/DVT is common in COVID-19, often without DVT. D-dimer thresholds from pre-COVID guidelines remain applicable.
9	Jackeline Hernandez-Nino et al. 2022 [20]	USA	Qualitative study of 24 VTE patient interviews	Provider communication (word choice, nonverbal cues) increased patient fear during VTE diagnosis. Incomplete information exacerbated anxiety.	Improved provider communication strategies are needed to reduce patient distress during acute VTE diagnosis in emergency settings.
10	Tai-Li Chen et al. 2023 [21]	Taiwan	Nationwide cohort study of VTE risk in atopic dermatitis (AD) (284,858 participants)	AD patients had higher VTE incidence (1.05 vs. 0.82 per 1000 person-years; HR 1.28). Risks were elevated for DVT (HR 1.26) and PE (HR 1.30). The absolute risk difference was small.	AD is associated with a modestly increased VTE risk. Cardiovascular evaluation may be warranted for symptomatic AD patients.
11	Fábio Henrique Rossi et al. 2024 [22]	Brazil	Perspective on severe VTE management (PERT® expansion to VTERT®)	Severe iliofemoral DVT requires aggressive treatment (e.g., thrombectomy) to reduce PE risk and sequelae. Multidisciplinary teams (VTERT®) improve outcomes.	A paradigm shift toward rapid, multidisciplinary response (VTERT®) for severe DVT/PE is recommended to improve acute and long-term outcomes.
12	Goran Koraćević et al. 2024 [23]	Serbia	Opinion piece on VTE terminology	Proposes renaming "pulmonary embolism (PE)" to "pulmonary thromboembolism (PTE)" to reflect thrombotic origin and align with DVT terminology.	Standardizing VTE terminology (DVT/PTE) could improve clinical and research clarity.
13	Qin Luo et al. 2024 [24]	China	Nomogram development for hospital-acquired VTE in cardiovascular patients (27,235)	Hospital-acquired VTE incidence: 0.57%. Risk factors included immobility, heart failure, and central catheters. The nomogram showed high accuracy (AUC 0.865 vs. Padua 0.786).	The nomogram provides a tailored, accurate tool for predicting hospital-acquired VTE in cardiovascular patients, outperforming existing models.

Dermatological Disorders and VTE

Dermatological diseases, particularly those with systemic inflammatory components, such as psoriasis and SLE, have shown a clear association with VTE [27]. As outlined in Table 3, these conditions often promote a prothrombotic state via cytokine-driven endothelial damage and platelet activation. In emergency settings, dermatological symptoms like livedo reticularis or purpura may be misinterpreted, delaying VTE diagnosis [28]. Interdisciplinary collaboration between dermatologists and emergency physicians is essential to recognize cutaneous markers of thrombosis and initiate anticoagulation therapy promptly [29].

**Table 3:** Risk factors and incidence of VTE in dermatological disorders CLE: Cutaneous Lupus Erythematosus, VTE: Venous Thromboembolism, HR: Hazard Ratio, CI: Confidence Interval, OR: Odds Ratio, AD: Atopic Dermatitis, SLE: Systemic Lupus Erythematosus, RR: Relative Risk

Dermatological Condition	Authors	Risk of VTE (HR/OR/RR)	Incidence Rate (per 1000 person-years)	Management Strategies	Clinical Implications
Cutaneous Lupus Erythematosus (CLE)	O Ahlehoff et al. 2017 [15]	HR 1.39 (95% CI 1.10–1.78)	1.20 (CLE) vs. 0.33 (reference)	Anticoagulation for high-risk patients; monitoring for thromboembolic complications.	Increased awareness of VTE risk in CLE patients; consider prophylactic measures.
Bullous Pemphigoid	Patompong Ungprasert et al. 2018 [16]	OR 2.69 (95% CI 1.79–4.05)	Not specified	Anticoagulation in high-risk cases; limited evidence for routine use.	Screening for VTE in bullous pemphigoid patients; further research is needed.
Atopic Dermatitis (AD)	Tai-Li Chen et al. 2023 [21]	HR 1.28 (95% CI 1.17–1.40)	1.05 (AD) vs. 0.82 (non-AD)	Cardiovascular examination for symptomatic patients; manage systemic inflammation.	Small absolute risk difference but warrants vigilance for VTE symptoms in AD patients.
Systemic Lupus Erythematosus (SLE)	O Ahlehoff et al. 2017 [15]	HR 3.32 (95% CI 2.73–4.03)	5.24 (SLE) vs. 0.33 (reference)	Aggressive anticoagulation; risk-benefit assessment for long-term therapy.	High VTE risk in SLE necessitates proactive management and patient education.

Pulmonary Disorders and VTE

Pulmonary diseases are intricately linked to VTE, particularly due to overlapping symptoms such as dyspnea, hypoxia, and chest pain [30]. Table 4 highlights the shared clinical manifestations of PE and exacerbations of conditions like COPD or interstitial lung disease. Misdiagnosis is common in emergency departments, often resulting in under-treatment or inappropriate therapies. Prompt use of imaging modalities (CTPA or Ventilation-Perfusion (V/Q) scans) and D-dimer testing is critical for differentiation [31]. Interdisciplinary management involving pulmonologists improves diagnostic accuracy and ensures the selection of optimal anticoagulation or thrombolytic strategies, particularly in high-risk patients.

**Table 4:** Risk factors and incidence of VTE in pulmonary disorders COVID-19: Coronavirus Disease 2019, ICU: Intensive Care Unit, VTE: Venous Thromboembolism, PE: Pulmonary Embolism, DVT: Deep Vein Thrombosis, LMWH: Low-Molecular-Weight Heparin, DOACs: Direct Oral Anticoagulants, CT: Computed Tomography, PERT: Pulmonary Embolism Response Team, VKAs: Vitamin K Antagonists, CTEPH: Chronic Thromboembolic Pulmonary Hypertension

Pulmonary Disorder	Authors and Study Year	Risk Factors for VTE	Incidence Rate	Management Strategies	Clinical Implications
COVID-19	Young Joo Suh et al. 2021 [19]	ICU admission, systemic inflammation, and immobility.	PE: 16.5%; DVT: 14.8% (meta-analysis).	- Prophylactic anticoagulation (LMWH/DOACs). - D-dimer monitoring (cutoffs: 500/1000 μg/L). - CT angiography for suspected PE.	High VTE risk in severe cases; D-dimer guides exclusion of PE but lacks specificity.
Pulmonary Embolism (PE)	Fábio Henrique Rossi et al. 2024 [22]	Iliofemoral DVT, postthrombotic syndrome, and chronic recurrence.	Leading cause of preventable in-hospital death.	- PERT teams (multidisciplinary rapid response). - Mechanical thrombectomy for severe PE/DVT. - Long-term DOACs/VKAs.	Aggressive intervention reduces mortality and chronic sequelae (e.g., CTEPH).

Cardiac Disorders and VTE

Cardiac disorders, especially atrial fibrillation, congestive heart failure, and myocardial infarction, both predispose to and are complicated by VTE [32]. Table 5 details how heart failure and arrhythmias increase stasis and endothelial dysfunction, fulfilling Virchow’s triad. VTE in cardiac patients is associated with increased morbidity, as it can exacerbate right heart strain or precipitate sudden cardiac death in the case of massive PE. Emergency management should include risk stratification tools (e.g., PESI score) and close cardiology consultation [33,34]. Cardiac patients require nuanced anticoagulant regimens, considering concomitant antiplatelet therapy as well.

**Table 5:** Risk factors and incidence of VTE in cardiac disorders VTE: Venous Thromboembolism, CVD: Cardiovascular Disease, LMWH: Low-Molecular-Weight Heparin, DOACs: Direct Oral Anticoagulants, ACS: Acute Coronary Syndrome, ICU: Intensive Care Unit, CT: Computed Tomography, PE: Pulmonary Embolism, DVT: Deep Vein Thrombosis, VKAs: Vitamin K Antagonists, CI: Confidence Interval, CTEPH: Chronic Thromboembolic Pulmonary Hypertension, Fuwai model: Clinical prediction tool developed in Fuwai Hospital, Warfarin: Vitamin K antagonist used for anticoagulation, Nomogram: Graphical calculating tool for individualized risk prediction

Cardiac Disorder	Authors	Risk Factors for VTE	Incidence Rate	Management Strategies	Clinical Implications
Hospital-Acquired VTE in Cardiovascular Patients	Qin Luo et al. 2024 [24]	Immobility, heart failure, central catheters, and obesity.	0.57% (hospitalized CVD patients).	- Nomogram-guided prophylaxis (Fuwai model). - LMWH for high-risk patients. - Early ambulation.	Tailored VTE prevention improves outcomes in CVD patients.
General Cardiovascular Diseases	Qin Luo et al. 2024 [24]	Female sex, age, infection, pulmonary hypertension, obstructive sleep apnea, acute coronary syndrome, cardiomyopathy, heart failure, immobility, central venous catheter, and intra-aortic balloon pump.	0.57% (hospital-acquired VTE)	Nomogram-based risk prediction (Fuwai model); anticoagulation for high-risk patients	Tailored VTE risk assessment is critical for cardiac patients due to overlapping risk factors (e.g., immobility, heart failure).
Heart Failure	John A. Heit 2015 [13]	Hospitalization, advanced age, immobility, and comorbidities (e.g., obesity, cancer).	Not explicitly quantified (elevated risk)	LMWH, warfarin, or DOACs based on guidelines	Heart failure patients are at high risk for VTE; prophylaxis is underutilized in clinical practice.
Acute Coronary Syndrome (ACS)	Walter Ageno et al. 2016 [14]	Immobility, central venous catheters, and post-surgical stasis.	Not specified (context-dependent)	Anticoagulation (avoiding triple therapy if possible)	ACS patients often develop VTE due to prolonged bed rest; balance bleeding/thrombosis risks.
Pulmonary Hypertension	Walter Ageno et al. 2019 [17]	Chronic immobility, right heart dysfunction, and central venous catheters.	10.1% had active cancer (VTE risk modifier)	DOACs are preferred over warfarin in non-cancer patients	Pulmonary hypertension exacerbates VTE risk; monitor for DVT/PE in symptomatic patients.
Post-Cardiac Surgery	Melanie Nana et al. 2020 [18]	Surgery, immobilization, obesity, and prior VTE history.	25,000 preventable deaths/year (UK estimates)	Multidisciplinary prophylaxis (e.g., "VTE stickers," education, early mobilization)	Hospital-acquired VTE is preventable; standardized protocols improve compliance.

Interdisciplinary Management Approaches

Effective VTE management in complex patients demands interdisciplinary teamwork. Table 6 summarizes emergency protocols across specialties, emphasizing early recognition, risk stratification, and standardized imaging and anticoagulation pathways. The introduction of Pulmonary Embolism Response Teams (PERT) and multidisciplinary rounds has significantly improved patient outcomes in high-risk VTE cases. Dermatologists, pulmonologists, cardiologists, hematologists, and emergency physicians must collaborate to avoid delays in diagnosis and reduce mortality.

**Table 6:** Diagnostic and communication challenges in VTE VTE: Venous Thromboembolism, DVT: Deep Vein Thrombosis, PE: Pulmonary Embolism, PTE: Pulmonary Thromboembolism

Study Focus	Authors	Key Challenges	Solutions/Recommendations	Impact on Patient Care
Unusual Site VTE (e.g., cerebral/splanchnic)	Walter Ageno et al. 2016 [14]	Lack of clinical trial evidence; bleeding risk in splanchnic VTE	Anticoagulation for cerebral VTE; delay if bleeding risk in splanchnic VTE	Tailored approaches reduce morbidity/mortality in complex cases.
Provider Communication at Diagnosis	Jackeline Hernandez-Nino et al. 2022 [20]	Fear due to word choice, incomplete information, and nonverbal cues	Structured communication training and balance reassurance with clarity	Poor communication exacerbates anxiety; improved dialogue aids adherence and trust.
Terminology Controversy (DVT vs. PTE)	Goran Koraćević et al. 2024 [23]	Ambiguity in "PE" (embolism) vs. "PTE" (thromboembolism)	Proposed shift to "PTE" to emphasize thrombotic origin	Standardization may improve clinical understanding and research consistency.

Strategic recommendations and clinical integration

Key strategies derived from this review include several measures aimed at improving the management and diagnosis of VTE. While this systematic review provides comprehensive insights into the interdisciplinary management of VTE in dermatological, pulmonary, and cardiac disorders, several limitations should be acknowledged. First, the inclusion of predominantly observational studies and a scarcity of randomized controlled trials (RCTs) may limit the strength of causal inferences. Second, heterogeneity in patient populations, diagnostic criteria, and treatment protocols across the included studies complicates direct comparisons and synthesis of findings. Third, the review relied solely on English-language publications, which may have excluded relevant data published in other languages. Furthermore, certain dermatologic and pulmonary conditions lacked extensive data, potentially underrepresenting their association with VTE. Limited reporting on long-term outcomes and adverse effects of anticoagulation in these patient populations also constrains the generalizability of management recommendations. Lastly, the absence of standardized definitions and risk stratification models specific to dermatologic VTE presentations highlights a gap in existing clinical tools. Future research should prioritize high-quality RCTs, focus on developing disease-specific VTE risk models, and emphasize interdisciplinary trials to validate integrated management strategies.

## Conclusions

This systematic review draws attention to the complexity and multiplexed nature of VTE in dermatological, pulmonary, and cardiac disorders, highlighting the need for timely recognition and cooperative management in the emergency environment. Inflammatory skin conditions, together with respiratory insufficiencies, cardiovascular disease, and VTE, not only exacerbate the risk for VTE but also compromise its correct identification and treatment. The findings emphasize the need for a multidisciplinary approach that includes dermatologists, pulmonologists, cardiologists, and emergency physicians to allow for timely prophylaxis, accurate risk stratification, and personalized therapeutic strategies. Moreover, increased awareness and the implementation of evidence-based guidelines are essential for improving outcomes, minimizing complications, and lowering recurrence rates, particularly in patients with comorbidities or those presenting with atypical symptoms. This review highlights the need for ongoing research and heightened clinical vigilance to address existing practice gaps and enhance patient care pathways at the intersection of these specialties.
